# Characteristics of good supervision: a multi-perspective qualitative exploration of the Masters in Public Health dissertation

**DOI:** 10.1093/pubmed/fdw107

**Published:** 2016-10-03

**Authors:** Srinivasa Vittal Katikireddi, Jacqueline Reilly

**Affiliations:** 1 MRC/CSO Social & Public Health Sciences Unit, University of Glasgow, Top Floor, 200 Renfield Street, Glasgow G2 3QB, UK; 2 Department of Public Health, University of Glasgow, 1 Lilybank Gardens, Glasgow G12 8RZ, UK

**Keywords:** education, educational settings, employment and skills, public health

## Abstract

**Background:**

A dissertation is often a core component of the Masters in Public Health (MPH) qualification. This study aims to explore its purpose, from the perspective of both students and supervisors, and identify practices viewed as constituting good supervision.

**Methods:**

A multi-perspective qualitative study drawing on in-depth one-to-one interviews with MPH supervisors (*n* = 8) and students (*n* = 10), with data thematically analysed.

**Results:**

The MPH dissertation was viewed as providing generic as well as discipline-specific knowledge and skills. It provided an opportunity for in-depth study on a chosen topic but different perspectives were evident as to whether the project should be grounded in public health practice rather than academia. Good supervision practice was thought to require topic knowledge, generic supervision skills (including clear communication of expectations and timely feedback) and adaptation of supervision to meet student needs.

**Conclusions:**

Two ideal types of the MPH dissertation process were identified. Supervisor-led projects focus on achieving a clearly defined output based on a supervisor-identified research question and aspire to harmonize research and teaching practice, but often have a narrower focus. Student-led projects may facilitate greater learning opportunities and better develop skills for public health practice but could be at greater risk of course failure.

## Background

The Masters in Public Health (MPH) was historically the first opportunity to gain the core knowledge and expertise demanded of the discipline,^1^ with a dissertation commonly required. Despite this, there is a lack of clarity about the purpose of the MPH dissertation and its necessity long questioned.^2^

The modern MPH reaches a range of students with varied disciplines and backgrounds—more so than was historically the case in the UK. This echoes the growing diversity within the public health workforce.^3–6^ The prior disciplines of students, therefore, now span the breadth of the arts, humanities, sciences as well as the world of healthcare.^3^ This increased diversity has allowed a genuinely inter-disciplinary and increasingly international approach which is a necessity for future public health practice and research.^7–9^

Despite the broad use of the MPH dissertation in many universities, there is limited research on the views of students and supervisors.^10–13^ Research is necessary since the higher education literature highlights the importance of subject and qualification level in influencing supervision and research–teaching linkages,^14–16^ with the Master's dissertation particularly regarded as an ill-defined ‘chameleon’.^17^ The pedagogical literature draws attention to the benefits of making the processes of postgraduate degree supervision explicit for both supervisors and students.^18^ Given the growing diversity of students served by the MPH, and the large number of supervisors, there is a risk that a shared understanding may be lacking. We explored the purpose of the MPH dissertation from the perspective of both students and supervisors, and identify practices viewed as constituting good supervision.

## Methods

To gain an in-depth understanding of the range of views, a multi-perspective qualitative interview study^19^ was undertaken with staff and students. This design explicitly allows diversity in participants’ views to be sought (including comparisons between staff and students). The stated purpose of the MPH dissertation at this institution is to provide an opportunity ‘to carry out an original piece of work’ and projects run from January to August annually. It could involve primary research, analysis of secondary data or a (semi-systematic) literature review.

Potential staff participants were chosen on the basis of their University website profiles, supplemented by snowball sampling. A purposive sample aiming for diversity of supervisor experience (senior staff and junior staff), parent discipline (clinical, social sciences and statistics) and methodological expertise (quantitative and qualitative) was sought. Potential participants were initially sent an information leaflet by e-mail and invited to participate, with a maximum of three e-mails in the case of non-response.

Purposive sampling of students sought diversity of disciplinary background (healthcare related, non-healthcare related), country of origin (UK, international student) and dissertation methodology (quantitative and qualitative). Students supervised by J.R. were ineligible for interview.

Informed consent was obtained at the interview and recorded in writing. Topic guides, informed by existing literature and advice from an expert in pedagogical research (see Acknowledgements), were created to help structure interviews, with coverage of core topics included in both staff and student interviews, but further questions tailored for each set of participants (see Supplementary Appendix). Staff interviews were carried out by S.V.K. (at the time, a public health specialist registrar who had not supervised MPH dissertations) and student interviews by J.R. (an MPH course university teacher who has supervised many students). All data were collected approximately midway through the dissertation period, so students were still accessible for interviews. Interviews were audio recorded and typically lasted 30–45 min.

Following verbatim transcription, interview data were read repeatedly and analysis proceeded in keeping with the principles of grounded theory.^20,21^ Inductive thematic coding was conducted by S.V.K. and J.R., with initial descriptive codes created and subsequently recoded to characterize emergent themes. The principle of the constant-comparative method was used to help identify explanations for patterns within the data while also paying attention to contradictory data.

The study was approved by the University of Glasgow College of Medicine and Veterinary Medicine research ethics committee.

## Results

Of the 10 staff approached, all agreed to be interviewed but a suitable time could not be arranged with 2, resulting in 8 staff participants (and good sample diversity achieved). Seventeen students were invited to participate and 10 interviewed, with the intended diversity achieved. No further descriptive details or disaggregation of quotations beyond ‘Supervisor’ or ‘Student’ are provided, to ensure anonymity.

Below, we present key emergent themes: first, briefly outlining interviewees’ views on reasons for undertaking the MPH; second, more detailed consideration of the MPH dissertation's purpose in particular; third, perspectives on dissertation supervision and finally, identified tensions that impact on the supervision process. In the Discussion, we build on these findings to develop two putative ideal types to describe alternative dissertation supervision approaches.

### The purpose of the MPH

Many participants’ views of the dissertation echoed their views of the MPH's overall purpose, with three main objectives highlighted. First, the MPH was seen as serving an important ‘credentialising’ role. Its acquisition could allow career progression or provide a gateway into the field of public health, but this required the MPH to be in ‘good standing’ so that its acquisition demonstrated a certain level of competence. Second, it provides an opportunity to acquire core disciplinary knowledge (such as epidemiology) and discipline-specific skills (e.g. critical appraisal). Lastly, there was an understanding that the degree provided practical training for public health practice, or less commonly, research. One interviewee neatly summarizes this:

Supervisor: And I know that some of the students come because it's part of their career progression. I think some of them are just really interested in it [public health] and it's a chance to be really interested in something for a year. I guess they're all looking to attain a recognisable qualification which marks them out as having a certain level of knowledge and perhaps some skill, some research skill. […] some of them are looking to get that then to get into public health.

Some participants perceived a tension between the preparing students for public health practice and the ivory tower of academia while others saw these as complementary:

Supervisor: Well I think traditionally it's [the MPH has] been a kind of broad-based preparation for the world of Public Health, for people to take up the types of jobs that they do in fact tend to take up once they graduate from here. So, while it's a fairly academic programme a lot of the posts in Public Health do tend to be fairly academic.

### The purpose of the dissertation

Table 1 summarizes key themes identified. There was broad agreement that the dissertation process allowed students to gain generic skills (such as writing, project management, time management and the ability to work independently) (Table 1a), as well as discipline-specific skills. Many respondents saw a clear relationship between taught courses and the dissertation process, with the dissertation providing an opportunity to apply knowledge and hence strengthen learning from taught courses (Table 1b). The dissertation was also seen as an important assessment method (Table 1c).

**Table 1:** The purpose of the dissertation

Theme	Illustrative quotation
(a) Acquisition of skills	Supervisor: Well, I think the main thing about a dissertation, I suppose this is pretty much the same as it is in any masters course to a large extent, in that it's providing an opportunity for people to… or a demand really for, not an opportunity, to work independently and work on a sizeable piece of work and work with less supervision than probably they've ever done before in relation to an academic type piece of work, and taking responsibility for completing it. And that is not too dissimilar to the type of thing you would be doing in a Public Health job.
(b) Application of taught courses	Student: Doing the dissertation when we did was really good because we had already been given classes on Principles and statistics and methods, all of which were useful in coming up with an idea of what to do it on and also on how to go about it. My dissertation used qualitative methods so the taught course on qual. was really good for me as we had to get ethics and everything and really think about what the best way to collect data was, so yes there are links with the taught courses.
(c) Assessment	Student: I think how it (is) assessed seemed really fair. There are two internal markers and an external so you get a good range of people looking at it—I can't see how else you could do it to be honest.
(d) Opportunity to bridge research-practice divide	Student: Yes, it was really good, my work is in [TOPIC BLANKED] and here was one which was totally perfect for me. I knew it would be useful after this degree and I could take home a lot of really good research experience and knowledge.
(e) The need for a practice-based dissertation	Supervisor: And that we would serve our students better if we made the project much more analogous, the type of investigation and report which service Public Health either in this country or abroad needs to address. So, there's a cleft between my own view and the departmental view… The cleft I was hinting at earlier is that those [public health] skills can be applied in very different ways and the rules by which we assess them will vary according to whether they are pragmatically trying to answer big Public Health questions in which we frame the question often more widely and accept some of the inexactitudes that whizz out from that. Or are we trying to be ‘pukka researchers’ in which case we get narrower and narrower questions, which in my view become less and less relevant to actual Public Health practice.
(f) Alignment of research and teaching	Supervisor: and think about getting a publication out of those supervision sessions with that student, not as a first off necessarily, but so that it is also forming, so the academic practice is therefore informing the academic process … how you should really align research and teaching much better.
(g) Impracticality of publishing following dissertation research	Supervisor: … but they're not going to get a merit or the distinction then yeah, you know, you're not going to suddenly take that MPH thesis and publish it very quickly. You know, it's going to require additional work and it's unlikely that additional work will come from the student, it would have to come from the supervisor and that, you know, that can be di… , that would be difficult.
(h) An end product focus	Supervisor: I think there are some people who focus on the end product. So they want people to have published papers. And I think there are people who, at different times, see Master's students as additional research assistants who will go into other projects and collect data, you know, for something bigger. And they might have something that they can write up, but they haven't probably done all of the setting it up and thinking about the questions and everything else.

While respondents acknowledged that most students would not conduct comparable future research, some saw striking similarity between public health practice and research (Table 1d). Others also saw the insight experienced from carrying out research as a way to foster improved long-term communication between academia and practice. An alternative view highlighted tailoring the dissertation to the practice environment (Table 1e) but other respondents cautioned that projects originating from public health practice were often ill-suited, tending to be too broad and not adequately rigorous. The risk that students may be expected to carry out too large a project, as a result of unrealistic employer pressure, was expressed but tempered by an appreciation that employers may reasonably expect some benefits if they have funded students.

Another much debated purpose, and less so students, was the potential for dissertation research to result in academic publications. At best, this was seen as helping align research and teaching responsibilities for supervisors while benefiting students by helping improve their skills and CV (Table 1f). However, potential benefits to science and the supervisor's career were not accepted uncritically with one supervisor commenting: ‘the reality is—I don't need extra low-grade publications’*.* While there was an acknowledgement that publication may constitute a ‘win-win’ , some interviewees felt it might be impossible to achieve as students (and supervisors) may not have the requisite time and patience to follow-up on dissertation work (Table 1g).

Others expressed concerns about encouraging students to publish or seeing it as a goal to be pursued. If a publication was being considered by the supervisor, it was felt this may limit the student's potential for learning as a narrow project predefined by the supervisor is more likely (Table 1h). In addition, it was felt to be a more amenable model for dissertations using already collected data; hence, of more relevance for some (primarily quantitative) research. Students may, therefore, be less likely to learn and gain experience in primary data collection, a skill perceived as valuable by some.

### Good supervision practice

Respondents felt there was considerable supervision variation, with diversity between individual supervisors believed to be greater than diversity between institutions. This was frequently viewed positively as students could choose a supervisor with similar interests to their own (Table 2a). The mutual choice of student and supervisor allowed subsidiary purposes of the dissertation to be more easily satisfied—for example, students interested in gaining a publication actively look for projects provided by high-profile supervisors most likely to offer publishable projects.

**Table 2:** Good supervision practice

Theme	Illustrative quotation
(a) Diversity of supervision practice	Supervisor: I mean, there are some people around here who are much better than I am at giving students projects that they know will get through and the students are not blind to all this you know, so they'll pick those supervisors who they know have got good projects and they know and can supervise them well in that and can make all that happen. So I think in the spectrum of attitudes that you'll be sampling, there'll be colleagues around here who will be more towards that side of the spectrum. And I think one of the things that's good about the department is that we have that spectrum and in a sense, that allows me to be the type of supervisor I am because if we were all like me, I might have to be more like them if you know what I mean.
(b) Ability to guide students through the dissertation process	Student: It was good to meet up initially and get a clear idea of what was going to happen, when and how.. that helped a lot because it all seems so massive at the beginning, you can't see how you are going to get to the end, but when it was all broken down into parts that made it easier.
(c) Pastoral support	Student: I would have a panic and then go and see [name of supervisor] and everything was alright again. He really made me feel safe and that I was progressing well… I think it's really important to be told that.
(d) Equity of supervision	Supervisor: I would like to think all the students I supervise get, you know, from me a similar amount of interest and I try to, you know, I'm equally invested in all of them. You know, I want them all to do really well, but they don't all … in order to achieve that, they don't all necessarily need the same approach. So, for some students, for example, I need to see them weekly, just because I know that that is what they require. Others, you know, they can go for a month and I know that when I see them in a month they'll have made lots of progress and they'll have interesting things to debate and discuss. And I don't see that as an inequality, I know some people in the department do see that, and would be horrified and are horrified that that goes on, but I see it my job is to deliver the best supervision I can for the student.
(e) Perceived unfairness of supervision	Student: Well [name of another student] was up there nearly every day and some people were like what's that all about, I know it's different depending on what you are doing but it can look a bit unfair when people don't really understand what has to go into the different projects … so I would say there was a little bit of discontent from some people.

Supervisors and students broadly agreed on a number of key elements for good supervision. First, it was felt necessary for supervisors to have good knowledge about what constitutes a dissertation and therefore be able to guide students through the process (Table 2b). Furthermore, having expert knowledge of the topic they were supervising and technical expertise on the research methods were viewed as important. While prior topic knowledge was not always considered essential, supervisors indicated that they would endeavour to learn about it so they could guide the student appropriately. Supervisors were expected to have several skills, including being organized (with accurate note-taking commonly recommended), clear communicators and able to provide pastoral support and encouragement if required (e.g. Table 2c). More specific suggestions about the conduct of supervision sessions included setting ground rules, providing timely and meaningful feedback and being available to students.

Supervision practice was often viewed as requiring a tailored approach which developed over time, based on student ability, with more directive feedback needed for less well-performing students and more high-level feedback required for students aiming for a distinction. It was acknowledged that this meant not treating students equally, but instead hopefully equitably (Table 2d). There was general agreement amongst supervisors that flexible supervision was important and strict rules on contact hours per student (as occurs in some MPH degrees) seen as unhelpful. However, the system of varied contact time was deemed potentially problematic by some students (Table 2e).

Supervisors’ reflections led to some advice for new supervisors. Amongst these was the need to remember that the project is the student's dissertation and not the supervisor's. It was also highlighted that supervisors would inevitably get better with experience but the budding supervisor should accept this as part of the process and forgive themselves for early mistakes.

### Pressures on the dissertation process

Supervisors frequently commented on the pressures impacting on the dissertation process. Following the growth in student numbers, and increasing diversity of students’ backgrounds (both in terms of disciplines and nationality), it was appreciated that assuring high-quality supervision for everyone could be challenging. This was echoed by the student perspective, with some international students noting the need for greater support to develop a ‘critical’ approach to reading academic literature (Table 3a).

**Table 3:** Pressures on the dissertation process

Theme	Illustrative quotation
(a) A need for greater support for some students	Student: I know I understand it but did worry that because everything is in English I was missing some important elements of different texts and was doing the critical analysis needed for the literature review well enough.
(b) Dissertations give students the opportunity to help develop research questions	Supervisor: Because I think it is important that research … my personal view is I don't think research questions should necessarily be framed by the supervisor right at the beginning. I think it's good to let the student have a part in developing what the research questions are. There might be a general idea from the supervisor but I think formulating research questions is something that the dissertation can help. A student can show off their skills in that.
(c) Students deriving research ideas as impractical	Supervisor: Some of my colleagues like all of their students to completely develop everything from a blank sheet of paper. I personally don't. I think if somebody comes to me with a well formulated idea that's fine but I think the majority of students aren't in a position to do that. To be honest if you're able to do that, you probably don't need to be on the MPH. You've probably already got a PhD. To have the proper level of understanding to know what's the right depth of research, a feasible project and the right way to do it methodologically is quite an advanced skill.
(d) Tension between research commitments and dissertation supervision	Supervisor: I am aware that because I'm very heavily involved in research I prefer to supervise students that are within my area of research interest which is a deliberate ploy to be efficient and I guess in an ideal world students could do whatever they like. I think within the department as a whole we offer that. We offer quite a range of people and we get some people who are more prescriptive than others and so on. But I think it is a slight self-protection mechanism in that if I were to supervise a large number of students doing a wide range of things and it involved a huge amount of legwork on my part, having to get to grips with a totally novel area and different methodologies, that's not an efficient use of my time and arguably I'm not the best person to supervise it.
(e) Challenges in assessing diverse dissertation types	Supervisor: The difficulty is that we are a mixed discipline and mixed experience department and we, all of us, set and mark the projects. Therefore, we've sought to get over the diversity of temperament and experience and background in what we mark by having ever-stricter criteria and these are most easily applied to quantitative traditional epidemiological studies and probably the systemic reviews where we have a well-established set of rules about what makes a good project. It's harder to apply to qualitative, purely qualitative studies although I think we've made some progress in defining what we see as good quality projects in that context. It's much harder to keep that agreed system of appraisal going in mixed method approaches and in narrative review approaches, or mixed method approaches informed by a narrative review, as would be the case in almost everything I've ever seen done in Scottish Government or in Health Boards or elsewhere and there is… in my mind an extraordinary paradox that we teach Public Health pretending that this kind of more pure approach will somehow be applicable.
(f) Practice-derived dissertations	Supervisor: Some of them have come with questions from their funders, if they're coming from the Health Board or from an organisation. That organisation might say, ‘we want you to do this piece of work,’ and that's often quite difficult, because it–sometimes it makes a good dissertation, often it doesn't. And you have to sort of work around that.

A tension was identified between students developing their own research topic and the need for supervisors to have some knowledge of the dissertation topic. Some supervisors felt it was preferable for students to play an integral part in conceiving the research question (Table 3b), while others felt this was unrealistic at the MPH level and within the dissertation timescale (Table 3c). Other priorities, especially research, were often seen as competing with dissertation supervision but some supervisors attempted to align these two priorities—exemplified by aiming for academic papers resulting from dissertations (Table 3d).

Tensions were identified between the dissertation as a credentialising tool and as a learning process. The former favours a standardized process which is amenable to clear marking guidelines. Within the department, attempts have been made to accommodate diversity in disciplinary approaches by having specific marking guidelines for different methodologies (such as systematic reviews and qualitative research). However, there was some criticism of this on at least two fronts (Table 3e). First, the validity of such guidelines and their ability to allow comparison of different forms of research was questioned. Second, the focus on the end product as a piece of research was felt to potentially limit opportunities for conducting more practice-orientated work (as carried out within government departments or elsewhere), which might be more relevant to a student's learning requirements but less easily definable as a specific form of research (Table 3f).

## Discussion

### Main finding of this study

**Fig. 1:**
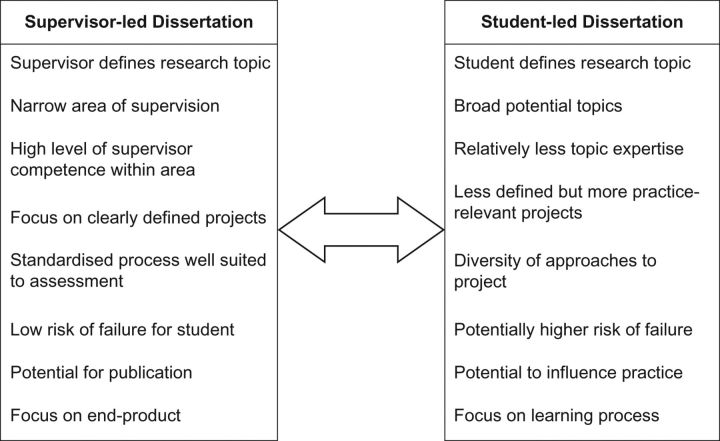
A representation of two ‘ideal types’ of the MPH dissertation process.

Students and supervisors generally agreed that the MPH dissertation serves several purposes, including providing an opportunity to develop skills, apply learning from taught courses and help prepare for future work. Supervision is often tailored to students’ evolving needs and while a number of behaviours facilitate basic competence, good supervision is to some extent learnt from experience. However, we identified tensions in the supervision process, with two ideal types discernible (see Fig. 1). Supervisor-led dissertations tend to be narrowly defined by the supervisor and well suited to the credentialising purpose of the dissertation. In contrast, a student-led dissertation is more tailored to public health practice and some students’ learning requirements. The latter may require greater supervisor effort and put the student at greater risk of failure when the end product is assessed against criteria for a research product. In reality, a broad continuum exists between these ideal types and they represent a negotiated process that unfolds over time, rather than equating to supervisors (who may tend to operate more in one mode than another but switch their practice depending on the project and student).

### What is already known?

Existing pedagogical literature supports some of the themes we identify including what constitutes good supervision practice (such as subject expertise and guidance on time management and writing) and having a student focus.^22,23^ A recent Dutch qualitative study of pedagogy identified the importance of Master's supervisors adapting to students’ needs, but not their expectations.^24^ Similar diversity in Master's in Medical Education projects has been previously found, as have tensions between service commitments for NHS staff and their postgraduate supervision roles, prompting the authors to call for less reliance on service staff.^25^

Views on the benefits of a research perspective for students appear mixed. Struthers *et al*. sought views from medical, veterinary and dental schools, finding that many academic staff felt research thinking and skills were important in informing professional practice.^26^ In contrast, Gabbay highlighted a perceived gulf between public health research and practice some time ago, arguing for experiential learning grounded in the real world.^27^ Much of the higher education research has focussed on whether research improves teaching quality but a meta-analysis found little relationship between the two.^28^ In contrast, qualitative research suggested that a complex interplay exists between research and teaching which varies by each individual academic.^16^

Achieving synergies across research and teaching is an academic priority in many institutions, with the publication of students’ research projects noted to be a potentially important way to encourage future researchers.^26^ In addition, public health academic departments have long had close relationships with practice—a strength which could be diminished as a result.^29^

### What this study adds?

By identifying the diverse expectations and needs of students, we hope supervisors are better able to match their supervision style to deliver the best possible learning experience and that our model assists in achieving this. Our study also suggests that a linkage between research and teaching is not without risk since academics may focus on one over the other.^14^ A research emphasis may result in public health practice skills being neglected.^3^ Our study goes beyond viewing research and teaching as in opposition or synergy. Instead, it points to a potential parallel to the posited ‘squeeze on intellectual spaces’—occurring when researchers have their academic freedom limited by the increasing focus on producing applied knowledge.^30^ Our findings raise the possibility that a comparable ‘squeeze on learning spaces’ may be occurring, where students’ freedom to explore and learn during the dissertation is curtailed—echoing a perceived decline in the intellectual environment experienced by postgraduate nursing students.^31^ This may result in MPH graduates finding it more difficult to bring together disparate research approaches in the manner often required for practice.

### Limitations of this study

This study investigated the topic of MPH dissertation supervision using qualitative interviews with supervisors and students, but several limitations exist. First, this is a small-scale study at a single institution. Further work is needed to establish the extent that these themes are evident elsewhere, including within more practice-oriented MPHs. That said, many respondents had experience of teaching elsewhere and supervisors felt dissertation supervision did not differ markedly between universities but more by supervisor. Second, while the interviewers’ institutional positions assisted in accessing interviewees, data obtained are influenced by our working relationships. For example, students may have been less open to voicing criticisms, particularly since the dissertation was ongoing. Lastly, while we have introduced a continuum of dissertation supervision types, this interpretation should be considered preliminary and further longitudinal studies to explore the evolving nature of supervision over time is needed.

## Conclusion

We report several findings worthy of reflection by new and experienced MPH dissertation supervisors alike. An awareness of the different purposes may assist supervisors to tailor their own and their department's supervision. Tensions identified in supervision raise questions about how academic public health departments could best respond to students’ changing needs. We hope such critical reflection of current pedagogical practice will assist in improving training for future generations of public health professionals.^32^

## Supplementary Material

Supplementary Data
