# Supplementary material for: Characteristics of good supervision: a multi-perspective qualitative exploration of the Masters in Public Health dissertation
Source: J Public Health (Oxf). 2016 Oct 3;39(3):625–32. doi: 10.1093/pubmed/fdw107 (PMC5939875; doi:10.1093/pubmed/fdw107)
Supplement: Supplementary Data [file fdw107_Supplementary_Data.zip › JPH_OnLineAppendixv1a.docx]

**Interview Guide for MPH Dissertation Supervisors**

Section 1 – Expectations of the MPH

1. What you think the purpose of getting a MPH is?
2. What role do you consider the MPH dissertation to have? Is it important and why? What learning do you expect students to get from their experience?
3. What types of research do you think are appropriate for a MPH dissertation? Are there specific things that are or are not suitable?
4. What do you think students want to get from the MPH in general, and from the MPH dissertation in particular?

Section 2 – Expectations of MPH Students

1. What do you expect from your MPH students when supervising?
2. What makes students particularly enjoyable or challenging to supervise?
3. How would your teaching practice adapt to the student’s ability?

Section 3 – Supervision Practice

1. What do you think students expect from their supervisor? What do you think they should expect?
2. What do you think makes a particularly good supervisor
   1. From the student’s perspective
   2. From the university’s perspective
   3. From your peers’ perspective
3. What makes particularly good or bad supervision practice? Can you think of any examples?
4. Do you think that supervision practice differs:
   1. Between supervisors
   2. Between universities
5. What would your advice be to new supervisors?

Section 5 – Concluding Questions

1. We’re almost at the end of the interview and I’d like to ask you whether you feel there is anything important we haven’t spoken about yet. Is there anything you would like to say?
2. Is there anyone you think I ought to contact in relation to this research?

Thank you very much for participating in this research.

**Interview schedule for MPH students**

Role of the MPH project

1. What role do you think the project has within the MPH?
2. What do you want to get from carrying out the research project?
3. At this stage do you think it will be helpful in your professional life? In what way?
4. What challenges do you think you will face in carrying out the work?

Project

1. What made you decide on the project you chose? What were the main issues in that decision/
2. Were you given choices of projects?
3. Were you able to offer a project of your own?
4. Did you discuss projects with potential supervisors prior to choosing?

Supervision

1. What do you expect from your supervisor at this stage?
2. Have you developed a plan of action for the research?
3. Is it clear to you what you have to do and how you will achieve it?
4. What level of supervision do you think you might need/want?
5. Are there any issues at this early stage that you think might need highlighting?

Completion of project

1. How did the project go for you? Was it a positive/negative experience?
2. Was the process what you expected? In what ways?
3. What were the main positives/ negatives of carrying out the research process? (What did you learn from the process?)
4. How did you find the supervision process?
5. Do you feel you were supervised effectively? (Yes/No? In what ways? Examples?)
6. What could have been done differently in your opinion?
7. Was your experience (in your view) different or the same as other students (in terms of supervision practice)? In what ways?
